# *OPRD1* SNPs associated with opioid addiction are *cis*-eQTLs for the phosphatase and actin regulator 4 gene, *PHACTR4*, a mediator of cytoskeletal dynamics

**DOI:** 10.1038/s41398-021-01439-y

**Published:** 2021-05-25

**Authors:** Orna Levran, Matthew Randesi, Miriam Adelson, Mary Jeanne Kreek

**Affiliations:** 1 The Laboratory of the Biology of Addictive Diseases, The Rockefeller University, New York, NY USA; 2 Dr. Miriam and Sheldon G. Adelson Clinic for Drug Abuse Treatment and Research, Las Vegas, NV USA

**Keywords:** Addiction, Clinical genetics

## Abstract

Several *OPRD1* intronic variants were associated with opioid addiction (OD) in a population-specific manner. This follow-up study aims to further characterize the *OPRD1* haplotype pattern of the risk variants in different populations and apply in silico analysis to identify potential causal variants. A population-specific haplotype pattern was revealed based on six *OPRD1* eQTL SNPs and five common haplotypes were identified in a sample of European ancestry (CEU). A European-specific haplotype (‘Hap 3’) that includes SNPs previously associated with OD and is tagged by SNP rs2236861 is more common in subjects with OD. It is quite common (10%) in CEU but is absent in the African sample (YRI) and extends upstream of *OPRD1*. SNP rs2236857 is most probably a non-causal variant in LD with the causal SNP/s in a population-specific manner. The study provides an explanation for the lack of association in African Americans, despite its high frequency in this population. OD samples homozygous for ‘Hap 3’ were reanalyzed using a denser coverage of the region and revealed at least 25 potentially regulatory SNPs in high LD. Notably, GTEx data indicate that some of the SNPs are eQTLs for the upstream phosphatase and actin regulator 4 (*PHACTR4*), in the cortex, and others are eQTLs for *OPRD1* and the upstream lncRNA ENSG00000270605, in the cerebellum. The study highlights the limitation of single SNP analysis and the sensitivity of association studies of *OPRD1* to a genetic background. It proposes a long-range functional connection between *OPRD1* and *PHACTR4*. PHACTR4, a mediator of cytoskeletal dynamics, may contribute to drug addiction by modulating synaptic plasticity.

## Introduction

Opioids act via the opioid receptors that have a major role in reward mechanisms, pain transmission, and drug addiction^1,2^. Opioid abuse is a major public health problem. The G-protein-coupled delta-opioid receptor (DOPr, DOR) is targeted by enkephalins and is widely distributed in the brain. It is involved in several brain processes, including learning and memory, anxiety, depression, and impulsivity^3^. It is also involved in regulating hyperalgesia as well as chronic inflammatory pain^4^. The opioid system has been well conserved throughout the evolution of vertebrates^5^. DOR is a target for the development of new pain therapies^6^. DOR is encoded by *OPRD1* (NM_000911.4) on chromosome 1 and translates a 372-amino-acid protein that is highly conserved in vertebrates.

Susceptibility to opioid dependence (OD) includes genetic and non-genetic components. Several *OPRD1* polymorphisms were associated with OD but many of the findings have not been replicated (for a recent review see ref. ^7^). Diverse genetic backgrounds and variation in linkage disequilibrium (LD) between populations may contribute to variable results in association studies. The *OPRD1* intronic SNP rs2236857 was associated with OD in our original association study of subjects of European descent (EA)^8^. This result was replicated in an independent Australian cohort^9^. SNP rs2236857 was also associated with response to life stress in subjects with OD in Han Chinese^10^. A second intronic SNP, rs2236861, was associated with OD in EA in our original study and this result was replicated in a study from Austria^11^. Association study of a cohort of African descent (AA) did not detect any association of *OPRD1* SNPs with OD^12^. One of the problems of association studies is that it is difficult to identify the causal variants since it is impossible to distinguish the signal of a marker from that of the other SNPs that are in LD with it. This challenge is greater when the LD structure differs between the populations. Non-causal markers may have different effects in different populations. Different approaches for fine mapping exist, including overlapping variants with functional elements^13^. Variants can disrupt the binding of transcription factors, resulting in a change in gene expression. Another approach is the use of quantitative trait loci (eQTL) information.

Our hypothesis was that the OD risk SNPs indicated in the original study are LD proxies for functional variant/s that are population-specific. To explore this hypothesis, we utilized publicly available functional and genetic data as well as data from our OD and control samples to obtain a higher resolution of the genetic structure of *OPRD1* and to provide an explanation for the results of the association studies.

## Materials and methods

### Sample

The sample includes subjects with opioid addiction (OD) and controls and was described in detail elsewhere^14^. Briefly, OD subjects (cases) were recruited at several opiate treatment programs in the US (e.g., Manhattan Campus of VA NY Harbor Health Care System, Weill Medical College of Cornell University, and Dr. Miriam and Sheldon G. Adelson Clinic for Drug Abuse Treatment and Research, in Las Vegas) or at the Rockefeller University. All cases had a history of at least 1 year of multiple daily uses of heroin and were on methadone maintenance treatment at the time of recruitment. The European American (EA) sample included subjects with >50% European/Middle Eastern ancestry contributions, based on structure or PC analysis as described^14,15^ (OD, *n* = 545; Controls, *n* = 196). The African American (AA) sample included subjects with >50% African ancestry contribution (OD, *n* = 308; Controls, *n* = 190); self-identified Hispanics were excluded.

The study was approved by the institutional review boards of the VA New York Harbor Health Care System and the Rockefeller University (for Rockefeller University and the Las Vegas clinic). All subjects signed informed consent for genetic studies.

### Genotyping

All samples were genotyped with the Illumina^®^ 1536-plex GoldenGate custom panel that included a limited number of *OPRD1* tag SNPs, as described^8^. A subsample was genotyped with the genome-wide custom Smokescreen^®^ array, as described^15,16^.

### Haplotype analysis and in silico functional analysis

Six SNPs were selected for haplotype analysis based on frequency, location, LD, and data from previous studies. Phased genotypes were obtained from the Ensembl genome browser. The main analysis was performed with the CEU (Utah residents with Northern and Western European ancestry) sample. The YRI (Yoruba in Ibadan, Nigeria), and CHB (Han Chinese in Beijing, China) samples were used for comparison.

The following databases were used for the analysis:

The Ensembl genome browser release 103 (www.ensembl.org)

IGSR: The International Genome Sample Resource (The 1000 Genomes Project) (https://www.internationalgenome.org/)

The Genome Aggregation Database (GnomeAD) V2.1.1 (https://gnomad.broadinstitute.org/)

The University of California, Santa Cruz (UCSC) Genome Browser (http://genome.ucsc.edu/)

The Allele Frequency Database (Alfred) (alfred.med.yale.edu)

LDlink (https://ldlink.nci.nih.gov/)

RegulomeDB (https://www.regulomedb.org/)

ENCODE (https://screen.encodeproject.org)

GeneCards (https://www.genecards.org/)

The Human Protein Atlas (https://www.proteinatlas.org/)

TarBase v.8, DIANA Tools (http://carolina.imis.athena-innovation.gr/diana_tools/web/)

The Genotype-Tissue Expression (GTEx)(https://gtexportal.org)

EMBL-EBI (https://www.ebi.ac.uk/).

## Results

### Selected SNPs for haplotype analysis

**Fig. 1:**
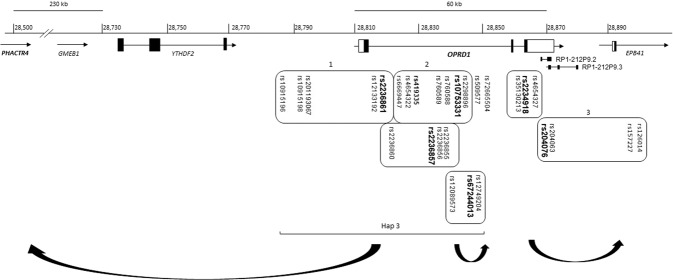
Schematic representation of the *OPRD1* gene region, SNPs locations, LD blocks, and target genes for eQTL. The selected SNPs used to build haplotypes are shown in bold. Open boxes represent LD blocks. Arrows connect eQTLs with their target genes.

Six SNPs were selected for haplotype analysis based on previous association studies, LD pattern, frequency, location, and potential functionality (Table 1). Based on GTEx, SNPs rs2236857 and rs2236861, indicated in our original association study of OD^8^, are the expression quantitative trait loci (eQTLs) for the phosphatase and actin regulator 4 gene, *PHACTR4*, located ~300 kb upstream of *OPRD1* (Fig. 1). The eQTL SNP rs10753331 is a proxy of SNP rs590013 that was associated with educational attainment^17^, as well as SNP rs419335 that was associated with OD^9^, and with decreased oxycodone analgesic response^18^. SNP rs67244013 is eQTL for *OPRD1* in the cerebellum and testis. The synonymous SNP rs2234918 (p.Gly307=) on exon 3 is one of the only two common coding *OPRD1* SNPs. It was indicated in several studies in association with OD, pain, and treatment outcome^10,19–21^, and is eQTL for *OPRD1* in the testis, and the erythrocyte membrane protein band 4.1 gene, *EPB41*, in the cerebellum. SNP rs2234918 is in high LD with SNP rs4654327, indicated in association with OD in Han Chinese^22^. The 3′ UTR SNP rs204076 is eQTL for *OPRD1* in the cortex.

**Table 1:** Selected SNPs for haplotype analysis.

	SNPs	Position Chr. 1 (GRCh38)	Location	Alleles^a^	Frequency of the variant allele			eQTL^b^	Association
					CEU	CHB	YRI		
1	rs2236861	28,785,050	Upstream	G>A	0.18	0.11	0.04	*PHACTR4*	OD, height
2	rs2236857	28,835,097	Intron 1	T>C	0.25	0.11	0.32	*PHACTR4*	OD
3	rs10753331	28,838,070	Intron 1	G>A	0.33	0.21	0.58^c^	*PHACTR4*	OD, analgesia
4	rs67244013	28,848,988	Intron 1	G>A	0.17	0.06	0.05	*OPRD1*, lncRNA ENSG00000270605	
5	rs2234918	28,863,085	Gly307=	C>T	0.61^d^	0.76^e^	0.28	*EPB41*	OD^e^, pain
6	rs204076	28,863,878	3′ UTR	A>T	0.34	0.13	0.14	*OPRD1, EPB41*	

*CEU* Utah residents with Northern and Western European ancestry.

*CHB* Han Chinese in Beijing, China.

*YRI* Yoruba in Ibadan, Nigeria.

^a^Ancestral allele > variant allele.

^b^GTEx, brain tissues.

^c^The variant A allele was the major allele in YRI and the minor allele in CEU and CHB.

^d^The variant T allele is the major allele in CEU and CHB and the minor allele in YRI.

^e^SNP rs2234918 is in high LD with SNP rs4654327, indicated in association with OD in Han Chinese^22^.

### LD blocks and cis-eQTLs

Several LD blocks, tagged by the selected SNPs, were identified in CEU (Fig. 1). They extend upstream and downstream of *OPRD1*. LD Block 1 extends from a region ~40 kb upstream to intron 1 of *OPRD1*, is tagged by SNP rs2236861, and includes several regulatory variants, including the regulatory SNP rs12133192. LD Block 2, on intron 1, is tagged by the regulatory SNP rs10753331 and extends ~21 kb. LD Block 3 is tagged by the 3′ UTR SNP rs204076 and extends ~30 kb from intron 2 of *OPRD1* downstream to *EPB41* (Fig. 1). The different LD pattern between populations affects the range of these LD blocks.

*OPRD1* is expressed primarily in the brain with high expression in the cortex, caudate, hippocampus, nucleus accumbens, and putamen. Moderate to low expression was shown in the cerebellum, hypothalamus, substantia nigra, and thalamus. There is a complex relationship between eQTLs and their target genes in the extended *OPRD1* region. While some *OPRD1* SNPs are cis-eQTLs for other genes, several SNPs in the *OPRD1* vicinity are *cis*-eQTLs for *OPRD1*. GTEx data indicate two distinct groups of eQTLs for *OPRD1*. The first group includes eQTLs for *OPRD1* in the cortex. This group is tagged by the selected 3′ UTR SNP rs204076 and is related to LD Block 3 (Fig. 1). These SNPs are also eQTLs for *EPB41* in the cerebellum, where it is highly expressed.

The second group represents eQTLs for *OPRD1* in the cerebellum. This group includes SNPs in *OPRD1* intron 1 and the upstream region. It includes SNP rs67244013 and its proxy SNP rs12749204 that are also eQTLs for an upstream lncRNA ENSG00000270605 (RP5-1092A3.4), as well as the regulatory SNP rs204048 that is eQTL for *PHACTR4* in the cortex. It also includes the *OPRD1* eQTL rs12089573. The significance of this group is undetermined since the function of *OPRD1* in the cerebellum is still unknown. Interestingly, lncRNA ENSG00000270605 is mainly expressed in the cerebellum, but its function is not known.

*OPRD1* is present in most vertebrates and its three exons are highly conserved. Comparative genomics data of the extended *OPRD1* region indicate a strongly conserved synteny of the *OPRD1* region on human chromosome 1, mouse chromosome 4, and rat chromosome 5. Notably, *PHACTR4* and *OPRD1* are adjacent in zebrafish whose common ancestors diverged ∼450 million years ago^23^. Zebrafish has two copies of *oprd1* on chromosome 19:14,921,000-14,951,756 (*oprd1a*) and on chromosome 16:34,160,835-34,174,260 (*oprd1b*), as a result of a known whole-genome duplication^5,24^. There are also two *phactr4* genes in these regions: *phactr4a* on chromosome 19, 191 kb from *oprd1a*, and *phactr4b* on chromosome 16, 25 kb from *oprd1b*.

### Major haplotypes

**Fig. 2:**
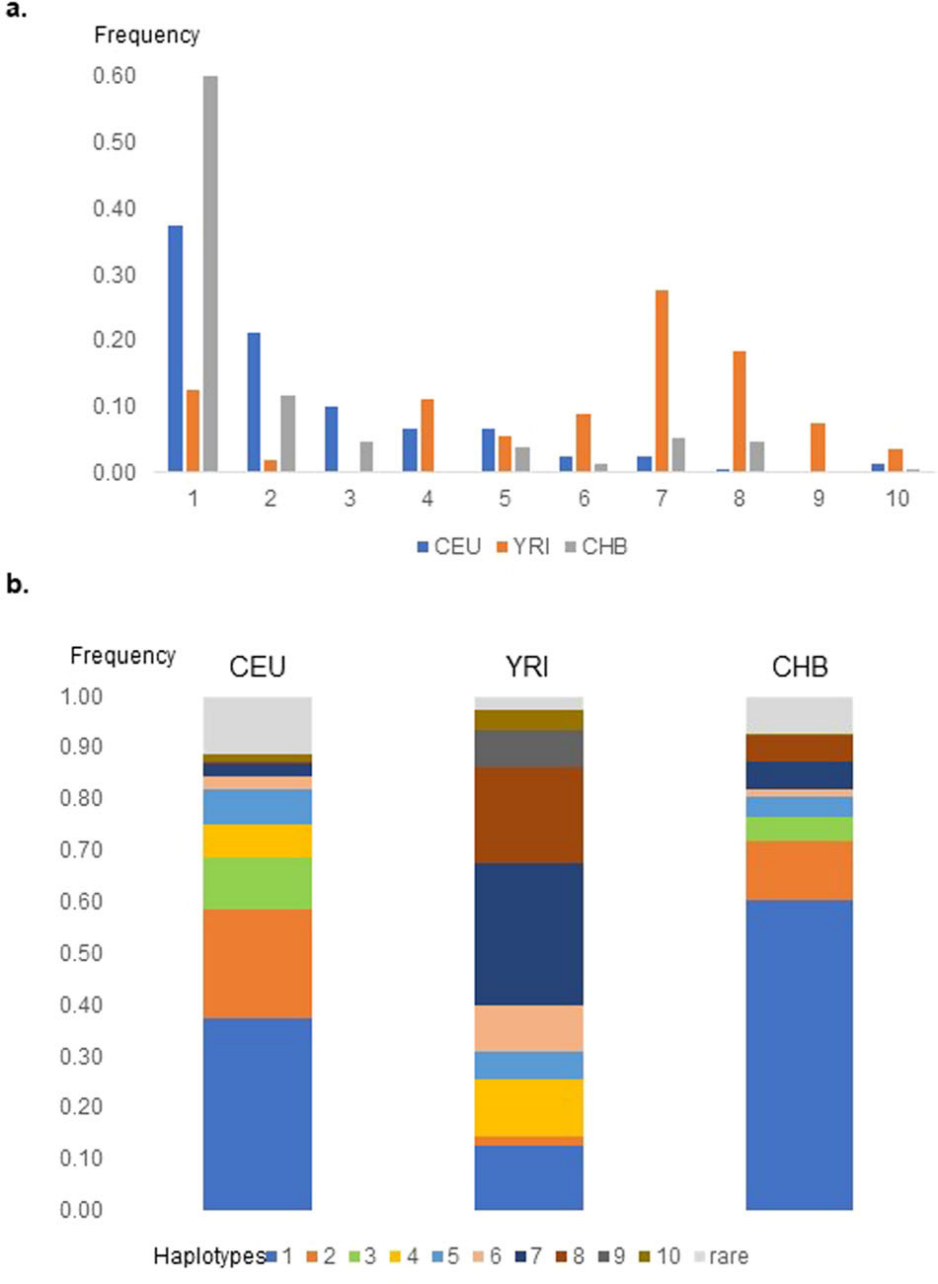
Distribution of major haplotypes in CEU, YRI, and CHB based on phased genotype data in three representative samples from the 1000 Genomes Project. **a** By haplotype, **b** By population.

Phased genotype data from the CEU sample were used to determine the major haplotypes from the selected SNPs and compared to representative samples from Asia (CHB) and Africa (YRI). Five major haplotypes (Haps 1−5) with a frequency of >0.05 were identified in CEU (Table 2 and Fig. 2). Hap 3 includes the variant C allele of SNP rs2236857, as well as the variant alleles of SNPs rs10753331, rs2234918, rs2236861, and rs67244013. It is uniquely tagged by rs2236861 among the common five haplotypes in CEU. SNPs rs67244013 and rs2236861 are redundant for defining the common haplotypes in this sample. Hap 3 is absent in YRI and is more common (10%) in CEU than in CHB (5%) (Table 2 and Fig. 2). It extends far upstream of *OPRD1* and includes numerous eQTLs for other genes in the region (e.g., *PHACTR4*). The variant C allele of SNP rs2236857 appears on two common haplotypes (Hap 3 and Hap 4) and several haplotypes that are rare in CEU and more common in YRI.

**Table 2:** Main *OPRD1* haplotypes.

	1	2	3	4	5	6			
Haplotypes	rs2236861	rs2236857	rs10753331	rs67244013	rs2234918	rs204076	CEU	YRI	CHB
**1**	G	T	G	G	**T**	A	0.37	0.13	0.60
**2**	G	T	G	G	C	**T**	0.21	0.02	0.12
**3**	**A**	**C**	**A**	**A**	**T**	A	0.10	0.00	0.05
**4**	G	**C**	**A**	G	C	**T**	0.07	0.11	0.00
**5**	G	T	**A**	G	**T**	A	0.07	0.06	0.04
6	G	**C**	**A**	G	**T**	A	0.03	0.09	0.01
7	G	T	G	G	C	A	0.03	0.28	0.05
8	G	T	**A**	G	C	A	0.01	0.19	0.05
9	G	**C**	**A**	G	C	A	0.00	0.07	0.00
10	**A**	**C**	**A**	**A**	C	A	0.02	0.04	0.00
Rare							0.11	0.03	0.07

Alleles in bold bases are variant alleles.

*CEU* Northern and Western Europeans from Utah, *YRI* Yoruba in Ibadan, Nigeria, *CHB* Han Chinese in Beijing, China.

There is a significant difference in the haplotype pattern between CEU and YRI and some similarities between CEU and CHB. The only common haplotype among the three samples is Hap 1. Haps 2 and 3 are rare in the African sample. Haps 7 and 8 are very frequent in YRI and rare in CEU and CHB.

This data highlights the higher resolution obtained by haplotype analysis compared to a single SNP analysis. The different pattern of haplotypes carrying the SNP rs2236857 variant allele in diverse populations and its appearance on more than one haplotype may explain in part the different association results in studies that were based on single SNPs. The intriguing finding that SNP rs2236857 was not associated with OD in African Americans despite its high frequency in Africa may be partially explained by the different haplotype patterns in different populations. It also suggests that SNP rs2236857 is not the causal SNP and may be a marker for a causal proxy SNP in a population-specific manner. Based on this information, association studies of *OPRD1* are sensitive to ancestry contribution. The LD between rs2236861 and rs2236857 differs between populations (*r*^2^ = 0.3 in CEU, *r*^2^ = 0.8 in CHB), as reflected in the haplotype architecture.

### Analysis of subjects with OD and controls

Subjects with OD and controls from two main ancestries (European and African Americans) were analyzed for *OPRD1* haplotypes. There was a higher frequency (6%) of homozygotes for the variant alleles of SNPs rs2236861 and rs2236857 (AA−CC) in the EA OD sample compared to controls (2%). There were fewer homozygotes for the reference alleles (GG−TT) in the EA OD (41%) compared to controls (48%), corroborating the original results. This SNP combination is rare in AA, as expected based on allele frequencies. The haplotype pattern of the EA and AA samples was similar to that of CEU and YRI, respectively (Table 2), except for Hap 3 that was at higher frequency in the OD EA group compared to controls and the CEU sample.

A selected region overlapping *OPRD1* was analyzed in-depth in samples that were homozygous for the main haplotypes and were genotyped by the genome-wide SmokeScreen^®^ array^15,16^. Additional information was obtained from the two CEU samples that were homozygous for Hap 3. Numerous informative high-quality SNPs were found to be carried specifically by the samples homozygous to Hap 3. Excluding SNPs in which the Hap 3 homozygous samples include the ancestor allele (e.g., SNP rs3766951), the emerging picture is of a long haplotype with at least 25 potentially regulatory SNPs (Table 3). The majority of the SNPs are in *OPRD1* intron 1 and some of them are shared by other less frequent haplotypes.

**Table 3:** Regulatory SNPs on Hap 3.

SNPs	Postion^a^	Location	Proxy of	RegulomeDB rank^b^	Probability	Regulatory type	eQTL *PHACTR4*	eQTL *OPRD1*	Additional major Haps in CEU
rs10915196	28,785,050	Upstream	rs2236861	4	0.61	CTCF binding site ENSR00000250915			
rs10915198	28,792,658	Upstream	rs2236861	3a	0.83	CTCF binding site ENSR00000250916	Yes		
rs201193067	28,792,782	Upstream	rs2236861	3a	0.81	Open chromatin ENSR00000921845			
rs12133192	28,812,628	Intron 1	rs2236861	2b	0.65	Promoter ENSR00000352105	Yes		
**rs2236861**	28,813,244	Intron 1	rs2236861	4	0.61	Promoter-like ENSR00000921847	Yes		
rs2236860	28,814,236	Intron 1	rs2236857	1f	0.55		Yes		4
rs6669447	28,822,849	Intron 1	rs10753331	3a	0.86		Yes		4, 5
rs4654322	28,824,733	Intron 1	rs10753331	1f	0.28		Yes		4, 5
rs419335	28,825,332	Intron 1	rs10753331	1f	0.73		Yes		4, 5
**rs2236857**	28,835,097	Intron 1	rs2236857	4	0.61		Yes		4
rs2236856	28,835,313	Intron 1	rs2236857	1f	0.55		Yes		4
rs2236855	28,835,487	Intron 1	rs2236857	1b	0.14		Yes		4
rs760589	28,835,953	Intron 1	rs10753331	1f	0.55		Yes		4, 5
rs760588	28,836,056	Intron 1	rs10753331	1f	0.55		Yes		4, 5
rs12089573	28,837,801	Intron 1	rs67244013	5	0.84		Yes	Cb	
**rs10753331**	28,838,070	Intron 1	rs10753331	1d	0.96		Yes		4, 5
rs2298896	28,839,626	Intron 1	rs10753331	2b	0.71		Yes		4, 5
rs509577	28,845,884	Intron 1		4	0.61	Promoter flanking region ENSR00000352114			
rs72665504	28,847,410	Intron 1		3a	0.75			Testis	
**rs67244013**	28,848,988	Intron 1	rs67244013	3a	0.87			Cb	
rs12749204	28,849,701	Intron 1	rs67244013	2b	0.73			Cb	

*Cb* cerebellum.

SNPs in bold are selecetd SNPs (Table 1).

^a^For indels only the upstream position is shown.

^b^The RegulomeDB probability score is ranging from 0 to 1, with 1 being most likely to be a regulatory variant. The RegulomeDB score represents a model integrating functional genomics features along with continuous values such as ChIP-seq signal, DNase-seq signal among others^39^.

Notably, based on GTEx data, the majority of the SNPs are eQTLs for reduced expression of *PHACTR4* in several brain tissues. Several SNPs are eQTLs for *OPRD1* in the cerebellum. Two regulatory SNPs (rs509577 and rs72665504) on intron 1 that have no proxies in CEU were identified as unique to Hap 3 based on these samples. SNP rs509577 is located in a regulatory region ENSR00000352114 and is an eQTL for several genes in non-brain tissues. SNP rs72665504 is an eQTL for *OPRD1* in testis.

Although no association of *PHACTR4* SNPs was reported with OD and the LD blocks that define the major *OPRD1* haplotypes do not extend to *PHACTR4*, we explored the relationship between two *PHACTR4* eQTLs (rs7518249 and rs4654365) and *OPRD1* haplotypes. There is low LD between SNPs rs7518249 and rs4654365 in CEU. The variant T allele of SNP rs7518249 is associated with reduced *PHACTR4* expression in several brain regions (e.g., *P* = 7.8e−13, caudate, GTEx). It is located in a functional CTCF binding site and promoter flanking region. SNP rs7518249 is in moderate LD (*r*^2^ = 0.33) with the *OPRD1* SNP rs2236861 in CEU. The variant G allele of the *GMEB1* SNP rs4654365 is also associated with the reduced *PHACTR4* brain expression (e.g., *P* = 1.0e−8, caudate, GTEx). SNP rs4654365 is in low LD (*r*^2^ = 0.13) with SNP rs2236861 in CEU. Haplotype analysis in our sample as well as in CEU revealed that the variant alleles of these *PHACTR4* eQTLs are part of Hap 3, although they are not unique to it.

Several SNPs that were reported in the literature were not included in this study for simplicity and technical reasons. SNP rs1042114 is a functional missense variant in exon 1 (c.80T>Gp.Phe27Cys). The minor frequency of the G allele is 13% in CEU and is very low in Africa and East Asia indicating that it has probably arisen after the split between European and Asian populations. In CEU, it appears mostly on Hap 2, sometimes on Hap 1, and on rare haplotypes that are independent of Hap 3. It is in complete LD with the upstream SNP rs61787581 located in a TF binding site and is also in high LD with several SNPs in intron 1, including the regulatory SNPs rs569356 and rs204051, as well as SNP rs533123 that was associated in a large-scale GWAS with schizophrenia^25,26^ and educational attainment^17^. SNP rs590013 that was associated with educational attainment^17^ is a proxy of SNP rs10753331 included in this study. SNP rs678849 was shown to predict OD treatment response in African Americans, but not in European Americans^21,27^. Hap 3 includes the ancestral C allele of SNP rs678849.

## Discussion

Association studies indicated *OPRD1* risk variants for drug addiction in a population-specific manner. Specifically, two intronic SNPs indicated in our original study in a cohort of European descent^8^ were replicated in independent samples of European ancestry^9,11^ but not in an African cohort^12^. These findings led to the hypothesis that these SNPs are LD proxies for functional variants in a population-specific manner. To explore this hypothesis, we have used publicly available data as well as data from our samples to define the haplotype structure of *OPRD1* and to perform in silico functional analysis.

Haplotype analysis suggested a large shift in the *OPRD1* genetic architecture between populations and showed the limitation of the original single SNP analyses. One of the target SNPs, rs2236857, was found to be a part of two common haplotypes in Europeans and two additional African-specific haplotypes. This population-specific pattern can explain the negative result obtained in the African American sample and highlights the sensitivity of association studies of *OPRD1* to population stratification. Notably, one of these haplotypes that is tagged by one of the original identified SNPs, rs2236861, in addition to SNP rs2236857, is quite common in European samples but is absent in African samples. The frequency of this haplotype was higher in the OD sample compared to controls, suggesting that it contributed to the association signal. In silico functional analysis revealed that SNPs within this haplotype are associated with a reduced expression for the upstream *PHACTR4*, encoding phosphatase and actin regulator 4, in the cortex. Additionally, some of the SNPs within this haplotype are eQTLs for *OPRD1* and an upstream lncRNA, in the cerebellum.

The study proposes a long-range functional connection between noncoding regions of *OPRD1* and *PHACTR4* expression that may provide an alternative explanation for the effect of OD risk variants. This connection was previously suggested, but not further explored^9^. This kind of long-range connection was shown in studies of other genes. For example, obesity-associated SNPs within the *FTO* gene that was known to be related to obesity were shown to form long-range functional connections with *IRX3*^28^. These findings have implications beyond these specific genes. It points to the need for careful interpretations of association studies, especially for noncoding risk variants. Long-range functional connections can be formed by long-range chromatin interactions that may skip over genes^29^.

### Opioid addiction, cytoskeletal dynamics, and synaptic plasticity

Neurons rely on cytoskeletal dynamics for synaptic plasticity. Synaptic plasticity is regulated by the polymerization of a cytoskeletal protein, actin. Actin cycling is controlled by transcription and epigenetic regulation of cytoskeletal proteins encoding genes^30^. Brain plasticity allows an individual to adapt to changes in the environment. Dendritic spines are dynamic post-synaptic structures that are thought to store memory. Drugs of abuse can alter actin dynamics and cause structural changes to dendrites, as was recently shown for the actin-binding protein, drebrin^31,32^. Drug relapse was associated with the involuntary retrieval of associative memories^33^.

The proposed association between *PHACTR4* and opioid addiction is intriguing*. PHACTR4* is part of a family of four structurally related members (*PHACTR1-4*)^34^. This phosphatase and actin regulator inhibits protein phosphatase-1 (PP-1), which is one of the main serine/threonine dephosphorylating enzymes in the cell^35^. Phactr4 was shown to have a role in a feedback loop that maintains actin monomers level^36^. Phactr4 was shown to regulate cytoskeletal remodeling during enteric neural crest cells^37^, and mice with a missense mutation in *Phactr4* had defective neural tubes and optic fissure closures^38^. *Phactr4* may have a different role in adult plasticity in addition to its developmental role.

In summary, the study suggested a large shift in the *OPRD1* genetic architecture between populations, identified a European-specific haplotype that is associated with OD, and showed the limitations of single SNP analyses. It proposes a long-range functional connection between noncoding regions of *OPRD1* and *PHACTR4,* and suggests that PHACTR4, as a mediator of cytoskeletal dynamics, contributes to drug addiction. If proven experimentally, this study can advance our understanding of the molecular mechanisms of synaptic plasticity in drug addiction and may provide an avenue for treatment.
